# Supplementary material for: Atomic-scale visualization of the interlayer Rydberg exciton complex in moiré heterostructures
Source: Nat Commun. 2024 Apr 22;15:3414. doi: 10.1038/s41467-024-47770-y (PMC11035671; doi:10.1038/s41467-024-47770-y)
Supplement: Supplementary file 1 — Supplementary Information [file 41467_2024_47770_MOESM1_ESM.pdf]

Supplementary information for Atomic-scale visualization of the interlayer Rydberg exciton complex in moiré heterostructures

Meng Zhao[1,2], Zhongjie Wang[1,2]*, Lu Liu[1,2,3], Chunzheng Wang[1,2], Cheng-Yen Liu[1,2], Fang Yang[4,5], Hua Wu[1,2,3,6†] & Chunlei Gao[1,2,4,5,6,7,8#]

1 State Key Laboratory of Surface Physics and Department of Physics, Fudan University, Shanghai 200438, China

2 Shanghai Qi Zhi Institute, Shanghai 200232, China

3 Laboratory for Computational Physical Sciences (MOE), Fudan University, Shanghai 200438, China

4 Institute for Nanoelectronic Devices and Quantum Computing, Fudan University, Songhu Rd. 2005, Shanghai 200438, China

5 Zhangjiang Fudan International Innovation Center, Fudan University, Shanghai 201210, China

6 Collaborative Innovation Center of Advanced Microstructures, Nanjing University, Nanjing 210093, China

7 Shanghai Research Center for Quantum Sciences, Shanghai 201315, China

8 Shanghai Branch, Hefei National Laboratory, Shanghai 201315, China

These authors contributed equally: Meng Zhao, Zhongjie Wang, Lu Liu

Table of contents

Supplementary Fig. 1: Computational partial density of states of sole monolayer $YbCl_3$.
Supplementary Fig. 2: Tip-interface distance dependency of the tunneling spectrum for in-gap excitonic states.
Supplementary Fig. 3: Moiré exciton complexes.
Supplementary Fig. 4: Topography and FFT images of different orientational domains.
Supplementary Fig. 5: Evolution of current-height characteristic from excitonic states to intrinsic band states.
Supplementary Fig. 6: Lateral distribution of $YbCl_3$ band states and Rydberg exciton LDOS.
Supplementary Fig. 7: I-z measurements performed on different samples.

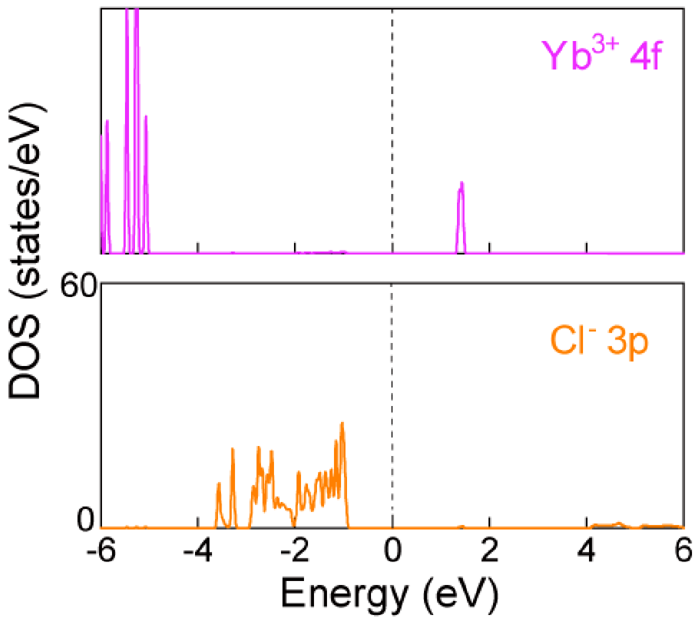

Supplementary Fig. 1 | Computational partial density of states of sole monolayer $YbCl_3$. The top panel illustrates the DOS of Yb-4f orbital, showing the upper Hubbard band (UHB) at 1.3 eV and the lower Hubbard bands (LHBs) from −9 eV to −5 eV. The bottom panel shows the DOS of Cl-3p orbital which constitutes the highest valence band. The overall DFT result demonstrates the electronic structure of charge-transfer insulator type of $YbCl_3$.

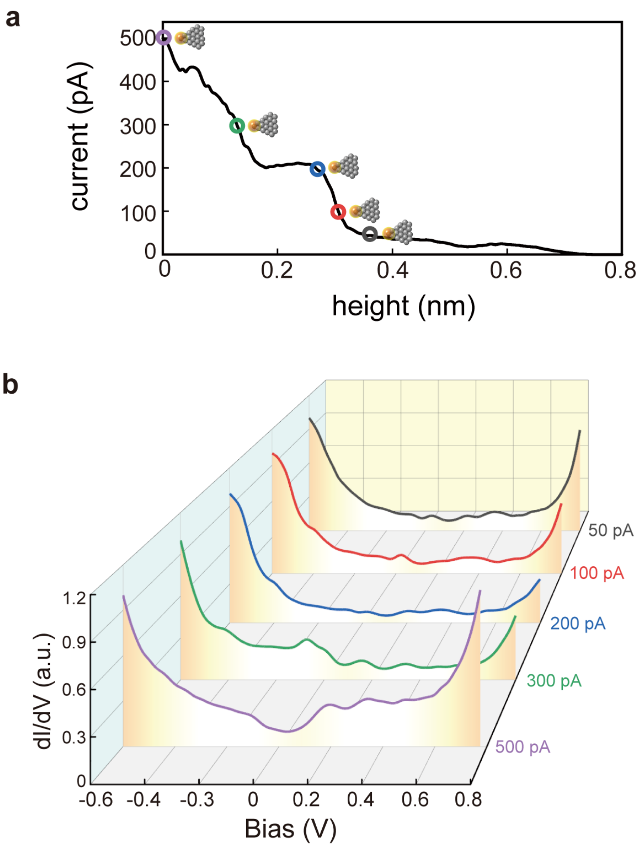

Supplementary Fig. 2 | Tip-interface distance dependency of the tunneling spectrum for in-gap excitonic states. **a** current-height curve taken at 0.7 V, the colored circles indicate the positions at which the tip is fixed and the tunneling spectroscopy is performed. **b** the corresponding dI/dV spectra taken at the positions marked in **a,** illustrating highly changeable features of the in-gap differential conductance. The legends indicate the setpoint current of each line. For clarity, all curves are normalized with respect to the dI/dV value at -0.6 V. As revealed by the current-height characteristics in Figs. 5c, 5d and 5e, the unusual shape of the current-distance curve can be decomposed into several Rydberg-like components. Each component dominates different spatial area along the out-of-interface direction. Changing the tip-interface distance is supposed to alter the relative ratio of Rydberg components that participate in the tunneling process, leading to a distinct dI/dV spectrum.

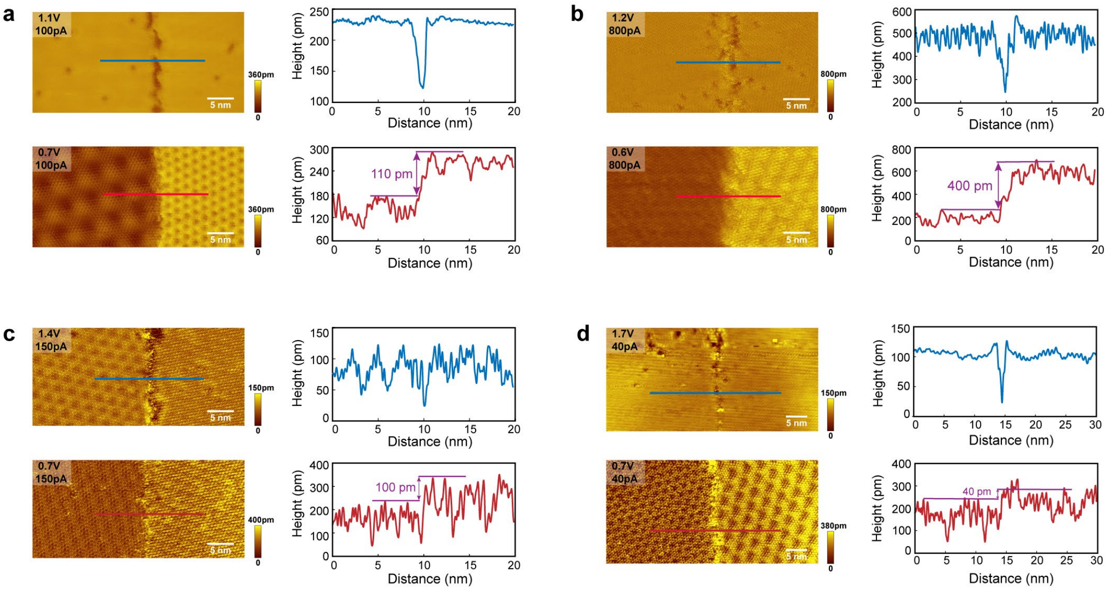

Supplementary Fig. 3 | Moiré exciton complexes. a constant-current STM images taken at the same sample area as displayed in Fig. 2d, but at a larger setup current of I=100 pA (the setpoint of each map is indicated). The height difference between the two domains observed in the bottom panel of **a** is about 1.1 Å, which is bigger than that of 0.4 Å in the right panel of Fig. 2d whose setup current is I=10 pA. **b-d** Images of other intersections of two domains with different superlattices. Similarly, no height difference is present in the image taken at the out-of-gap bias range, while remarkable height differences appear in the images taken at the in-gap-range bias. The corresponding setup conditions are indicated at the top-left boxes.

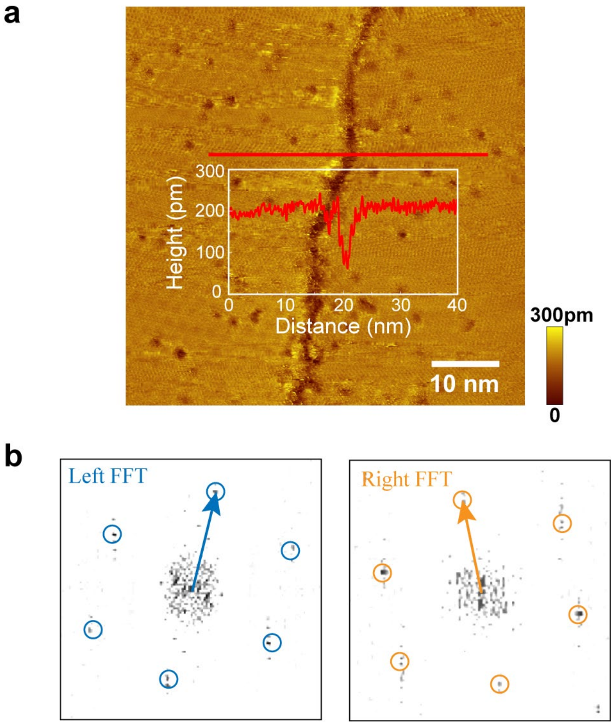

Supplementary Fig. 4 | Orientational domains. a STM image corresponding to the sample area of Fig. 3a with the imaging condition of U=1.1 V, I=100 pA. **b** the fast-Fourier transform maps obtained from the left and right domains, respectively. The blue and orange rings label the reciprocal spots of $YbCl_3$ lattice, illustrating the different lattice orientations of two domains.

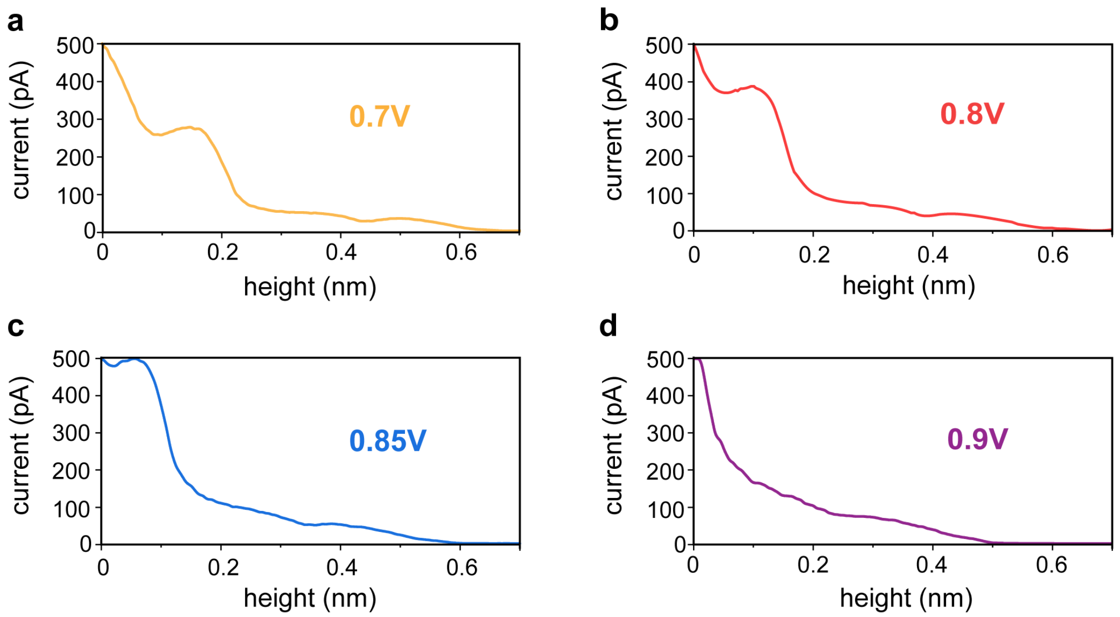

Supplementary Fig. 5 | Evolution of current-height characteristic from excitonic states to intrinsic band states. **a-d** current-height curves taken at different bias and different start points, the latter is determined by the corresponding setup condition: **a-d** share the same setup current I=500 pA, and the respective setup bias is indicated in the pictures. **a-d** show that, when increasing the tip-sample distance and tunneling bias to probe the intrinsic band states of $YbCl_3$ monolayer, as expected, a common monotonic decay is observed in the current-height characteristic (**d**).

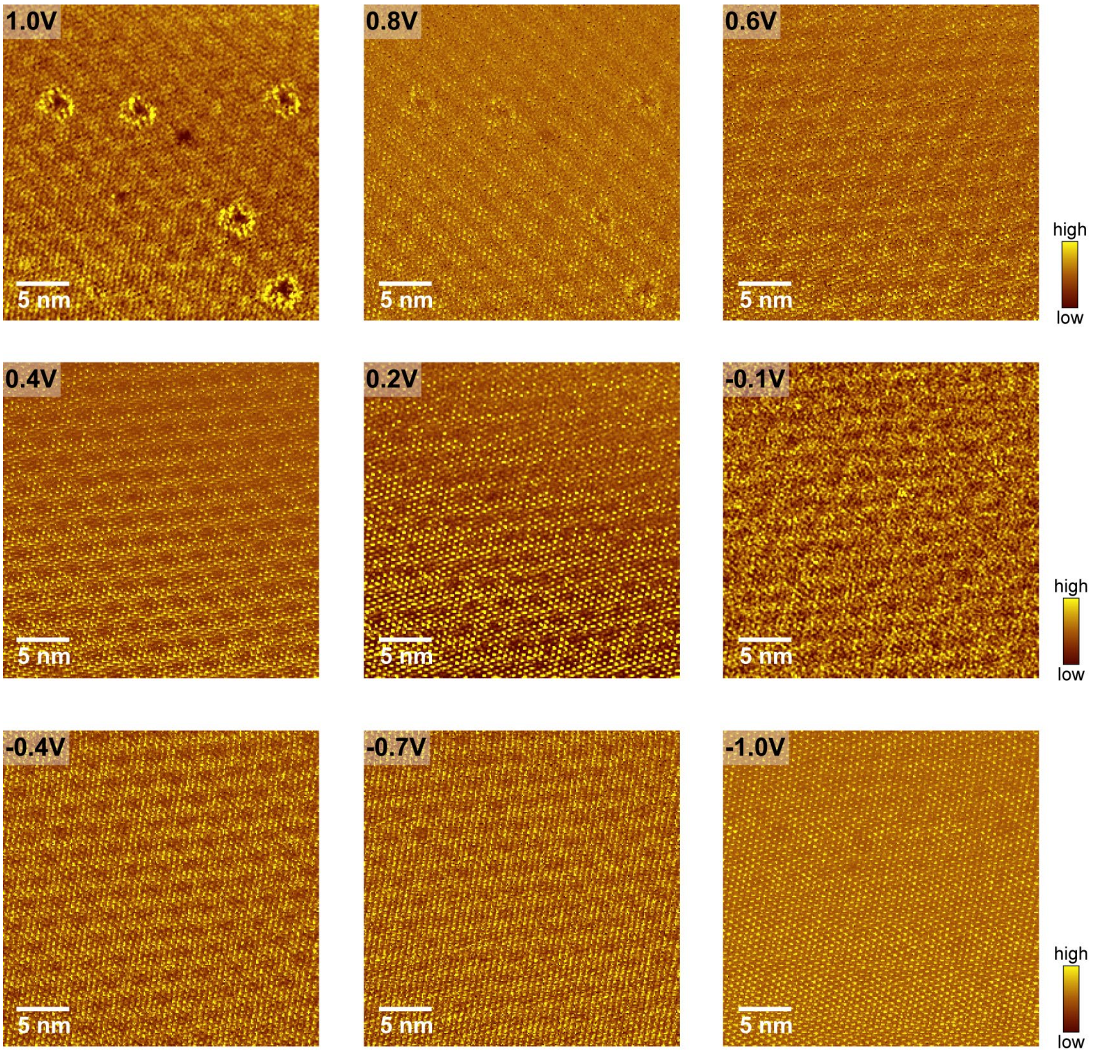

Supplementary Fig. 6 | Lateral distribution of $YbCl_3$ band states and Rydberg exciton LDOS. The images are a series of dI/dV maps extracted from a group of constant-height mapping data. The tip height is determined by the initial setpoint of U=1.0 V, I=100 pA. Then the feedback system is disabled, and the tip moves in a constant-height plane where the dI/dV spectra are taken for every pixel. The images demonstrate the lateral distribution of LDOS at corresponding energy where the Rydberg states show accordant in-plane distribution determined by the moiré periodicity. The tip current-position crosstalk effect is eliminated in the constant-height mode.

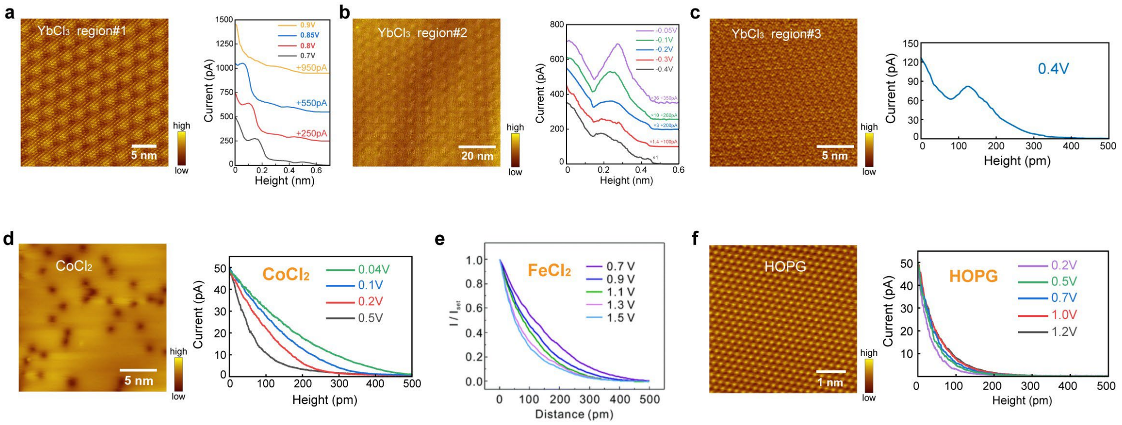

Supplementary Fig. 7 | I-z measurements performed on different samples. a-c I-z measurements performed on different regions of monolayer $YbCl_3$ with different tips. **d** I-z curves taken on another sample we studied, the monolayer $CoCl_2$ on HOPG. **e** I-z curves taken on the monolayer $FeCl_2$ on HOPG, which is extracted from our previous study[34], where the bias-dependent behavior is explained. Reprinted figure from Ref. [34] with permission of Royal Society of Chemistry. **f** I-z curves obtain on HOPG. Corresponding measuring biases are indicated in the picture.
